# Determination of the optimal method for measuring malondialdehyde in human saliva^✰^

**DOI:** 10.1016/j.mex.2024.103070

**Published:** 2024-11-23

**Authors:** H. Brignot, C. Rayot, G. Buiret, T. Thomas-Danguin, G. Feron

**Affiliations:** a Centre des Sciences du Goût et de l'Alimentation, CNRS, INRAE, Institut Agro, Université de Bourgogne, F-21000 Dijon, France; b Centre Hospitalier de Valence, 179 Boulevard du Maréchal Juin, 26953 Valence, France

**Keywords:** Lipoperoxidation, Oxidative stress, MDA, TBARS, Fluorescence, saliva, Using TBA derivatization and fluorescence

## Abstract

While few methodological studies have been published on the salivary measurement of malondialdehyde (MDA), none have precisely detailed the analytical method. This work presents the development of an analytical method for MDA measurement in microvolumes of saliva samples from healthy individuals, using thiobarbituric acid derivatization and fluorescence reading of the formed compound. This method was progressively designed to meet specific constraints such as the limited sample volume available, cost-effectiveness of each assay, time required for analysis and costs. After studying the impact of various parameters, quantitative validation of this method was conducted using the accuracy profile. The study demonstrated that the fluorometric analysis method for salivary MDA accurately quantifies the concentration of this molecule within a validity range from 0.031 μM to 2.3 μM. Additionally, the accuracy profile allowed estimation of measurement uncertainty for each concentration level. It was determined that the calibration method using raw data was most appropriate as it introduced the least uncertainty in the obtained results.

## Graphical abstract

**Figure:**
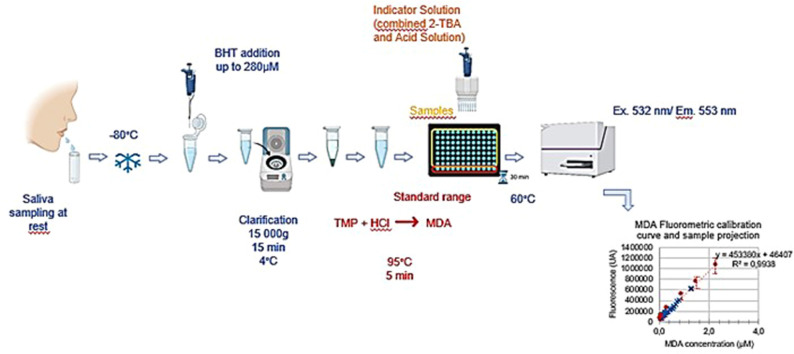

Specifications table

**Table:** 

Subject area:	Biochemistry, Genetics and Molecular Biology
More specific subject area:	Analytical biochemistry, Human Body Fluid, Sample Handling
Name of your method:	Using TBA derivatization and fluorescence
Name of your protocol:	Evaluation of malondialdehyde (MDA) levels in salivary human samples
Reagents/tools:	Analytical balance sensitive to 0.1 mg Fluorimetric plate reader capable of exciting the sample at 532 nm and reading at 585 nm. Polypropylene microcentrifuge tubes Deionized Water (dH2O) Adjustable monochannel and multichannel micropipettes (10 – 1000 μL) and tips Heated bain-marie Magnetic stirrer and magnetic bars Classic laboratory glassware 100 mL Reagent reservoirs Non-treated 370 µL working volume 96-well flat bottom black polypropylene wells Chemicals: TCA: SIGMA ALDRICH T9159–100 G
	NaOH: FLUKA 71,692 HCl: CARLO ERBA 403,871 Ethanol RPE: CARLO ERBA 4,146,072 TBA: SIGMA ALDRICH T5500–25 G BHT: SIGMA B1378–100 G TMP: SIGMA 108,383–100mL
Experimental design:	This paper describes an iterative method for the development of a protocol aiming at evaluating MDA levels in salivary human samples considering specific constraints (e.g. size of the sample). The following steps were conducted: - literature survey & choice of a fluorometric methodological approach - development of the protocol: choice of the wavelength, incubation time and temperature, reagent orders and concentrations, reagent stability, conditions for measurement - salivary sample handling (clarification, deproteinization) - characterization and validation using the accuracy profile
Trial registration:	*NA*
Ethics:	***As our work involved human subjects,****all the relevant informed consent was obtained from the volunteers*
Value of the Protocol:	• This protocol is valuable for obtaining an accurate estimation of MDA levels in saliva • This protocol is well suited for small sample size and for important number of samples in term of time consumed and cost • This protocol can serve as a tool to monitor oral lipid peroxidation in cancer patients

## Background

The perception of a metallic taste is a frequent occurrence in oncology, affecting 29 % of patients in a recent meta-analysis [1]. There are several possible explanations for the metallic taste, all of which may occur at the same time: lipoperoxidation, inhibition of the facial nerve on the glossopharyngeal nerve, presence of sweeteners, calcium and magnesium salts, anodal stimulation of the tongue, sectioning of the tympanic cord, direct stimulation of the tympanic cord, phantogueusia during pregnancy, burning mouth syndrome, metals in the mouth (iron, copper, zinc), etc. [2,3].. Among these potential causes, the peroxidation of lipid membranes of the oral cavity cells and salivary lipids, also called lipoperoxidation, could be a principal cause of the perception of this metallic flavor and discomfort in the mouth [[4], [5], [6]]. Lipoperoxidation refers to the oxidation of lipids in the presence of oxygen. It is one of the main biomarkers of oxidative stress [[7], [8], [9], [10], [11], [12]]. It concerns lipids with at least two carbon-carbon double bonds [13,14]. Polyunsaturated lipids are the preferred targets of free radicals due to the high vulnerability of the methylene group (-CH2), positioned between the double bonds.

The most studied class of molecules resulting from lipid peroxidation are α,β-unsaturated aldehydes. The decomposition of hydropexoxides results in the formation of carbonyls including malondialdehyde (MDA), 4-HNE (4‑hydroxy-2-nonenal), and 4-hydroxyalkenal (4-HAE), MDA being the major and stable final compound of lipid peroxidation [7].

The aim of this report was to develop a reproducible MDA analysis methodology, adapted to salivary samples. This methodology will be used in a study currently being carried out aiming at investigating the association between the presence of a metallic flavor and the amount of salivary MDA in cancer patients. On the basis of a literature search, a protocol was developed. Tests were carried out to vary the assay conditions, adapting to the constraints of sample micro-volume, the time allocated to the assay for use in future analysis campaigns, and the cost of the method.

### Description of protocol

This assay is generally part of a set of marker assays based on the same saliva sample collected from humans.

The direct calibration method has been chosen for the routine assay of salivary samples because of the low sample volume required, the latter usually being low-volume samples from clinical studies.

ABBREVIATIONS

MDA = Malondialdehyde

4-HNE = 4‑hydroxy-2-nonenal

4-HAE = 4-hydroxyalkenal

TBA = Acide Thiobarbiturique

TBARS = Thiobarbituric Acid Reactive Substances

BHT = 2,6-di‑tert‑butyl‑4-methylphenol

TMP = 1,1,3,3-Tetramethoxypropane

TCA = Trichloroacetic Acid

HCl = Chlorhydric Acid

NaOH = Sodium Hydroxyde

IQC = Internal Quality Control

UA = Arbitrary Units (data from fluorometric measurement)

PRINCIPLE OF THE PROCEDURE

**Fig. 1:**
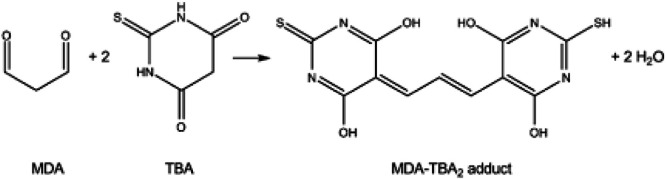
Formation of the fluorescent compound.

**Fig. 2:**
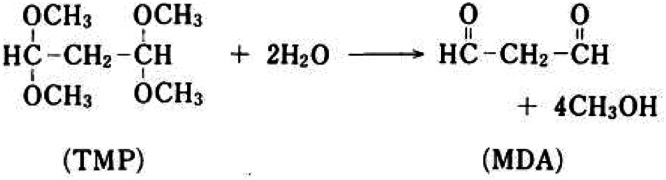
Transformation of TMP into MDA by acid hydrolysis (mole-by-mole reaction).

The principle of the assay is based on the reaction between one molecule of MDA and two molecules of TBA in an acidic medium at high temperature, resulting in the formation of a fluorescent product (ex.532 nm/em.553 nm), as shown in Fig. 1, Fig. 2. To avoid artifactual MDA formation during the procedure, an antioxidant (BHT) is added to the reaction medium. Heating during incubation enables determination of total MDA, i.e. MDA present in free and bound form. Calibration of the measurement is performed using a range of MDA, a substance manufactured extemporaneously by acid hydrolysis of a solution of TMP.

MATERIALS NEEDED

1. Analytical balance sensitive to 0.1 mg
2. Fluorimetric plate reader capable of exciting the sample at 532 nm and reading at 585 nm.
3. Polypropylene microcentrifuge tubes
4. Deionized Water (dH2O)
5. Adjustable monochannel and multichannel micropipettes (10 – 1000 μL) and tips
6. Heated bain-marie
7. Magnetic stirrer and magnetic bars
8. Classic laboratory glassware
9. 100 mL Reagent reservoirs
10. Non-treated 370 µL working volume 96-well flat bottom black polypropylene wells
11. Reagents :

**Table:** 

REAGENTS	N °CAS	REFERENCE
HCl	7647–01–0	CARLO ERBA 403,871
TMP	102–52–3	SIGMA ALDRICH 108–383
TCA (4 °C)	76–03–9	SIGMA ALDRICH T9159–100G
NaOH	1310–73–2	FLUKA 71,692
TBA	504–17–6	SIGMA ALDRICH T5500–25G
BHT	128–37–0	SIGMA B1378–100G
Ethanol absolute anhydrous - RPE - For analysis -ACS	64–17–5	CARLO ERBA 4,146,072

STORAGE OF PREPARED REAGENTS

1. BHT solution : 4 h
2. 1,25 % TBA solution in 0,3 % NaOH : 2 days at 4 °C in dark
3. 9,8 % TCA solution: 8 h
4. TMP solutions : 8 h
5. Reagent mixture (« F Solution ») : 4 h

WARNINGS AND PRECAUTIONS

1. Wear gloves and safety glasses when performing this assay, as the acid used is corrosive.
2. Work with Biological Safety Cabinets to handle biological samples to protect the user and prevent him from being contaminated by eventual pathogeneous from the sample but no during the protocol steps with acid chemicals handling.

PROCEDURAL NOTES

1. A sample from a saliva pool (a mixture of saliva from several individuals), aliquoted and stored at −80 °C, is introduced into all the plates, enabling inter-plate repeatability to be checked. It constitutes the IQC.
2. Samples, IQC and blanks should be run together on the same plate using the same standard curve.
3. Each sample is assayed in at least two replicates, and the concentration values obtained are averaged to determine the concentration of each sample. Each replicate is placed on a different plate. Example: if there are 3 plates, plate 1b is the replicate of plate 1, plate 2b the replicate of plate 2, etc.

REAGENT PREPARATION

1. 0.3 % NaOH solution: Weigh 1.5 g sodium hydroxide and dissolve in 500 mL ultrapure water.
2. Solution of 1.25 % TBA in 0.3 % NaOH: Weigh 312 mg thiorbarbituric acid and dissolve in 25 mL 0.3 % NaOH. Stir until the compound is completely dissolved.
3. 1 M HCl solution = 36.5 g/L: In a 10 mL flask, add 0.829 mL (i.e. 0.9865 g) of 37 % HCl solution and make up to the mark with ultrapure water.
4. 9.8 % TCA solution (for the reagent and a final concentration of 150 mM in the reaction medium): Prepare on the same day, 0.98 g in 10 mL pure water
5. BHT solution 3.5 mM = 0.771 mg/mL: Prepare the same day, weigh out 15.42 mg BHT and dissolve in 20 mL 99 % ethanol.
6. Reagent mixture: Solution F (for one plate): Mix 7.5 mL of 9.8 % TCA solution + 5 mL of 1.25 % TBA solution, then add 1 mL of 3.5 mM BHT solution to a beaker.
7. Standard range: MDA is prepared in the laboratory on the day of the assay from a commercial 6 M TMP solution.

This preparation is then successively diluted to obtain two solutions at 6 and 0.6 µM, which are used to produce the range directly in the microplate.

a. Pre-dilution of TMP stock solution (6 M):

Solution A (60 mM): 100 µL commercial TMP + 9.9 mL pure water
Solution B (1 mM): 200 µL of *A* + 11.2 mL ultrapure water + 600 µL 1 M HCl in a water bath at 95 °C for 5 min in a beaker.
Solution C (60 µM): 1.2 mL *B* + 18.8 mL pure water in a 20 mL flask

b. Dilution of MDA solution for calibration range:

Solution D (6 µM): 2 mL C + 18 mL pure water in a 20 mL flask
Solution E (600 nM): 2 mL *D* + 18 mL pure water in a 20 mL flask

SAMPLE STABILITY

1. Samples should be frozen at −70 °C/−80 °C to prevent loss of MDA and HAE and sample oxidation. Samples should not be stored at −20 °C.
2. Samples should not be refrozen several times and should be protected from light to avoid photooxidation.

SAMPLE PREPARATION

1. Thaw untreated saliva sample (whole saliva) at room temperature
2. Add BHT: 12 µL of 3.5 mM BHT for 150 µL of sample (i.e. 1 well)
3. Clarify this mixture in a 1.5 mL eppendorf tube (15 min at 15,000 g at 4 °C), recover the supernatant and freeze at −20 °C overnight, or perform the assay extemporaneously.
4. Internal Quality Control (IQC) preparation: The salivary pool sample undergoes the same preparatory steps as the samples.

STANDARD CURVE PREPARATION

1. MDA is prepared in the laboratory on the day of the assay from a commercial 6 M TMP solution.
2. This preparation is then successively diluted to obtain two solutions at 6 µM (D) and 600 nM (E), which are used to produce the assay directly in the microplate (see REAGENT PREPARATION SECTION).
3. The standard range and the reaction mixture are made directly in the microplates.

ASSAY PROCEDURE

1. Add ultra-pure water
2. Add salivary samples and IQC with multichannel pipette
3. Add MDA (solution D or E following Table 1)

**Table 1:** Fluorometric standard curve preparation.

MDA Conc. (μM)	nmole MDA/well	MDA Solution	V_H2O_ (µL)	V_MDA_ (µL)	V_SOLUTION F_ (µL)	V_TOTAL_ (µL)
0	0	–	165	–	135	300
2.10^–2^	0,006	E	155	10		
6.10^–2^	0,018	E	135	30		
1.10^–1^	0,03	E	115	50		
3.10^–1^	0,09	D	150	15		
9.10^–1^	0,18	D	135	30		
15.10^–1^	0,3	D	115	50		
22,5.10^–1^	0,45	D	90	75
4. Then add solution F (TBA+TCA+BHT) using a multichannel pipette, mixing back and forth 5 times.
5. Cover plate with cling film and aluminum foil, incubate 30 min at 60 °C
6. Stop the reaction by placing the microplate on ice for 10 min.
7. Remove adhesive film from microplate
8. Read fluorescence at λexcitation: 532 nm / λemission: 553 nm within 10 min

**Table:** 

	1	2	3	4	5	6	7	8	9	10	11	12
A	sample	sample	sample	sample	sample	sample	sample	sample	sample	sample	sample	sample
B	sample	sample	sample	sample	sample	sample	sample	sample	sample	sample	sample	sample
C	sample	sample	sample	sample	sample	sample	sample	sample	sample	sample	sample	sample
D	sample	sample	sample	sample	sample	sample	sample	sample	sample	sample	sample	sample
E	sample	sample	sample	sample	sample	sample	sample	sample	sample	sample	sample	sample
F	sample	sample	sample	sample	sample	sample	sample	sample	sample	sample	sample	sample
G	sample	sample	sample	sample	sample	sample	sample	sample	sample	sample	sample	sample
H	0	0.006	0.018	0.03	0.09	0.18	0.3	0.45	*0*	*CQI*	*CQI*	–

**Scheme 1:** Sample Plate Layout if 2 plates in replicate.

CALCULATIONS

1. Fluorescence values (in UA) obtained for all duplicated wells.
2. Do not subtract the fluorescence value of the Blank from the Standard and Sample fluorescence value.
3. Plot a standard curve using the fluorescence values (in UA) for each Standard versus the MDA concentration for each Standard by least squares linear calibration to calculate the equation of the linear model.
4. Check the instrumental response linearity of the assay: R² > 0.98
5. Calculate the equivalent MDA concentration expressed in µM equivalent MDA for each Sample using the fluorescence value and the equation generated by the Standard Curve.
6. Assay quality control.

Expected performance in calculated concentration (eq MDA) of the standard range and the IQC (salivary pool) within the concentration range:

a. Repeatability is defined as a measurement from the same assay same day and same reagents, intra and inter-plate
b. Reproducibility is defined by a measurement from different assays conducted in different days with different reagent preparations
c. The sample concentration value is valid if the fluorescence value is within the measuring range, i.e. for this assay: between the fluorescence value of the point at 0.006 and that at 0.09 nmol/well of MDA equivalent to a concentration from 0.02 µM to 2.3 µM MDA. Otherwise, if the value is not within the measuring range, dilute the sample 2 times if the value is above the upper limit and repeat the assay under the same conditions. If the value is below the lower limit, indicate 0.02 µM as the result.

**Table:** 

Reproducibility conditions		Variations (%) on concentration values calculated from 5 measurments	
		Standard range	Saliva samples
Repeatability	Intra-plate Intra assay	<10 %	<15 %
Repeatability	Inter-plate Intra assay	<15 %	<20 %
Reproducibility	Inter assay	<30 %	<30 %

### Protocol validation

**Fig. 3:**
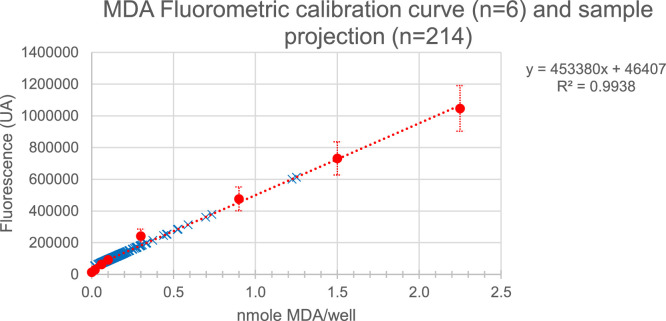
Typical Standard Curve (red dots) and normal and pathological samples projection (blue cross).

The Fig. 3 presents the results of the calibration curve and sample projection.

### Limitations

**Fig. 4:**
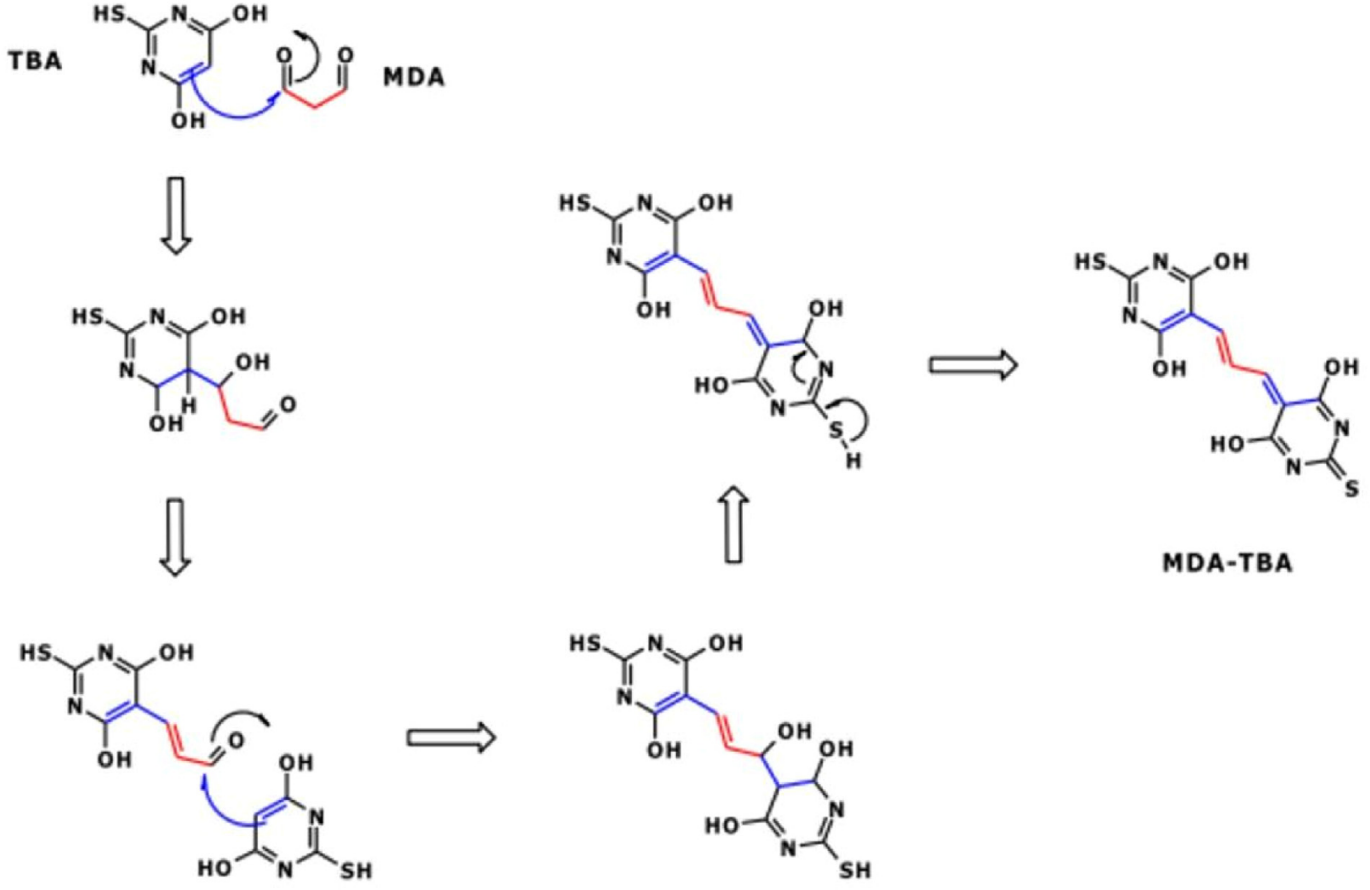
mechanism of the reaction between TBA and MDA.

The chosen method for total MDA measurement is the method using TBA, with detection conducted through fluorescence. Upon addition of TBA in the presence of MDA, the addition of two TBA molecules onto the MDA is observed [6,15,16]. The mechanism of the reaction between TBA and MDA is provided in Fig. 4.

The fluorescence of the formed complex can be measured at 553 nm (emission) and 532 nm (excitation) [17].

It is important to note that this method allows for the measurement of TBARS and not solely MDA. It is non-specific because TBA can react with other molecules [18]. Indeed, as discussed in the previous section, saliva contains numerous molecules that could interfere with the measurement of salivary MDA [19], such as other oxidation products like 4-HNE and HAE. Furthermore, the challenging experimental conditions (high acidity + high temperature) can lead to additional oxidation of available biological compounds in the sample, resulting in an overestimation of MDA quantification [19].

However, despite controversies regarding the specificity of TBA towards compounds other than MDA, it remains the most widely used method as an index of lipid peroxidation.

Moving forward, it is acknowledged that MDA is measured with the inherent imprecision it entails.

Several methods can be employed to control these artifacts:

- Since saliva contains numerous molecules capable of reacting with TBA, it is necessary to precipitate the lipoprotein fractions to minimize the formation of interfering substances. The use of precipitating agents enables the assay to be specific to MDA [20].
- Additionally, the use of BHT (an antioxidant) helps prevent additional formation of MDA during the assay [21].

The effect of storage over 18 months, a common timeframe in the context of research during clinical studies, has not been evaluated.

## Conclusion

The iterative approach implemented for designing the most efficient protocol for the evaluation of MDA levels in saliva was successful regarding the different constraints, i.e. size of the salivary samples, costs, and time consumed and despite some limitations described above. It will be used for investigating MDA levels in a clinical study conducted on cancer patients. However, this protocol could be also used for other studies with same aims.

## CRediT author statement

**Hélène Brignot:** Conceptualization, Methodology, Software, Validity tests, Data curation, Supervision, Writing, Original draft preparation. **Camille Rayot:** Conceptualization, Methodology, Software, Validity tests, Data curation. **Guillaume Buiret:** Validity tests, Data curation, Writing, Original draft preparation. **Thierry Thomas-Danguin and Gilles Feron:** Visualization, Supervision.

## Declaration of competing interest

The authors declare that they have no known competing financial interests or personal relationships that could have appeared to influence the work reported in this paper.

## Data Availability

I have shared my data with the link:

## Appendix Supplementary materials

Image, application 1
