# Supplementary material for: Determination of the optimal method for measuring malondialdehyde in human saliva
Source: MethodsX. 2024 Nov 23;14:103070. doi: 10.1016/j.mex.2024.103070 (PMC11732503; doi:10.1016/j.mex.2024.103070)
Supplement: Supplementary file 1 [file mmc1.docx]

**Supplementary material *and/or* additional information**

The supplementary material shows the progressive experimental approach used to create the protocol.

1. Literature review

Thirty-seven articles were identified (6, 15-50), describing a methodology for MDA level analysis. MDA levels specific to salivary medium were determined in only two studies (49, 50).

MDA was usually determined by derivatization, i.e. MDA forms an adduct with the derivatization molecule, which is then determined by various methods. Of the various derivatization molecules, thiobarbituric acid (TBA) was the most commonly used (23 out of 41 articles). Assay can be performed using a chromatographic separation method, or optionally using a traditional extraction method such as liquid-liquid extraction. The easiest of these methods use direct detection by spectrophotometry and fluorescence. As TBA is used as the derivatization molecule, they are therefore called TBARS (thiobarbituric acid reactive substances) methods. The hot acid condensation reaction between TBA and MDA leads to the formation of a pink pigment by Knoevenagel-type condensation. Fluorescence reading is possible on this molecule because it has several fluorophore groups: 2 conjugated aromatic rings and several π-bonds (double bonds).

Chromatographic analyses make the method more specific for MDA, as they enable the latter to be specifically selected from a number of end products formed during the free-radical-mediated decomposition of polyunsaturated fatty acids. Furthermore, saliva contains few other oxidation products that would react with TBA, compared with blood products, for example. However, these methods are more expensive than microplate-reader assays, due to the cost of the associated specific equipment, are more time-consuming and often require a high sample volume, ill-suited to saliva samples. In addition, pre-analytical liquid-liquid extractions consume a lot of sample amount and are likely to modify the initial content of oxidation products, so this type of preparation was not chosen.

Finally, detection by fluorescence is the most sensitive compared to detection by ultraviolet and visible spectrophotometry (37, 51). It was therefore decided to develop a method, which would enable us to assess lipid peroxidation in saliva and on oral cavity surfaces.

1. Development of the fluorometric assay method

All the assays described in the following paragraphs were carried out in black-walled and black-bottomed 96-well microplates (Thermo Fisher Scientific, Waltham, MA, USA), due to the constraints of micro-volume and fluorescence usage respectively. Fluorescence intensity data were acquired using the Ensight® plate reader (Perkin-Elmer, Waltham, MA, USA).

Parts 2.1 to 2.9 of the protocol development were carried out on model solutions composed of pure MDA, produced extemporaneously. Parts 2.10 to 2.12 were carried on human saliva from 10 healthy volunteers.

- 1. Initial version of the protocol

The initial version of the protocol was as follows: an MDA calibration range was established from the acid hydrolysis of a 1,1,3,3-Tetramethoxypropane solution (TMP). A reagent solution containing a 1.25% solution of TBA, a 50% solution of trichloroacetic acid (TCA) and a 3.5 mM solution of the antioxidant butylated hydroxytoluene (BHT), identified as the most efficient in this reaction (52), was added as a reaction mixture. The various concentrations of the range points were prepared directly in the plate, then the reaction mixture was added. The plate was filmed and incubated for 30 minutes at 60°C in an oven. To stop the reaction, the plate was then cooled in crushed ice. It was confirmed that the reaction in the absence of TBA did not generate an artifact.

- 1. Choice of emission wavelength and control of signal origin

The emission wavelength where fluorescence intensity was maximal was determined by plotting the emission spectrum of the TBA:MDA adduct with a known excitation wavelength, λexc = 532 nm and a range of emission wavelengths studied from λem = 550 to 580 nm with 1-nm-steps.

- 1. Study of the effect of incubation time and temperature

In the literature, incubation times ranged from 15 to 60 minutes and temperature ranged from 60 to 100°C. Studies also highlighted that there was a tendency to avoid over-oxidation phenomena during the assay reaction at 60°C compared to higher temperatures. Based on this, the incubation time was varied at 30 minutes and 60 minutes and the incubation temperature at 60°C and 80°C. The effect was estimated via a change on the calibration curve parameters.

- 1. Study of the effect of reagent introduction order

The aim was to study the order of introduction of reagents on the right-hand equation of a calibration range to control the effect of compound auto-oxidation.

In the initial protocol (§2.1), the reagent was prepared by mixing TBA, TCA and BHT together. In an alternative protocol, the calibration range was established by first mixing the MDA standard solution with BHT and TCA in the same proportions as in the initial protocol reagent. The plate was then incubated for 15 minutes at 60°C. The TBA solution was then added and the plate incubated again for 30 minutes at 60°C.

- 1. Study of the effect of BHT concentration

In the initial protocol, the BHT concentration in the reagent was set to 5%. We varied this BHT concentration to 0.5% and 10% to assess the effect on the equation of the straight line of a calibration range.

- 1. Determining the acid to use for the reagent solution

The reaction is only possible in an acidic environment (35). In the literature, around ten different acids have been used (37, 53), most often the same as those used for the preliminary extraction of the analyte. We have decided to compare only the two most frequently cited and common laboratory acids: TCA and acetic acid, whose pH has been reduced to 3.5 using NaOH.

- 1. Study of the effect of TCA concentration in the reaction medium

As the pH of the reaction medium had to be between 2 and 4 (35), it was necessary to control pH to ensure that the reaction takes place under the best possible conditions. In the initial protocol (§2.1), a 50% TCA solution was chosen in accordance with the literature describing the same assay applied to mitochondrial-type samples (41). During our experiments, a reaction pH of 0.35 before incubation and 0.12 after reaction was measured. To achieve the correct pH range which has to be between 2.5 and 4.5, the concentration of the TCA solution used for the reagent was lowered to 9.8% (52, 53).

- 1. Reagents stability

Assessment of reagent stability enabled us to evaluate the degradation of solutions over time, and to optimize the time allocated to each assay, by knowing which solution could be prepared in advance and which could not. To this end, we focused on TBA only and not on BHT. Indeed, the function of BHT is to prevent further oxidation when the reaction medium is heated and the choice was to not take any methodological risk regarding this preventing effect. For TBA solution, dosing was performed with the same solution from D to D+5.

- 1. Checking the MDA ranges of measurement

The initial version (§2.1) was based on a protocol conducted on mitochondrial suspension samples (41). However, in our study, the saliva used to develop the method came from 10 healthy subjects (with no pathologies or damage to the oral cavity) and the amount of MDA was supposed to be very low. For this reason, it was necessary to work with several concentration ranges. Two of the MDA ranges of measurement were used to set up the Protocol and are presented in this article.

- Range from 0 to 0.09 nmol/well

The range was set between 0 and 0.09 nmol MDA/well, corresponding to MDA concentrations ranging from 0 to 0.3 μM.

- Range from 0 to 0.18 nmol/well

Subjects treated for cancer have higher salivary MDA concentrations than healthy subjects, 1.0±0.21 µM vs 0.08±0.07 µM respectively (8). Although our method was developed on salivary samples from healthy subjects, it will be used for assaying samples from cancer patients. The study by Kaur et al (54) showed that the salivary MDA concentration of a cancer patient is equal to 1 µM, corresponding to an amount of 0.3 nmol/well, the test being conducted on 150 µL of saliva.

The range was thus set between 0 and 0.18 nmol/well, corresponding to MDA concentrations ranging from 0 to 0.6 μM. The aim was to ensure that the method's results were linear at concentrations close to the target concentration, and to enable modeling of the accuracy and uncertainty of results at these concentrations.

In addition, measurements were performed at concentrations between 0 and 0.006 nmol/well, to test the sensitivity of the assay. Finally, the protocol was developed to be able to quantify 2.3 µM equivalent MDA into saliva to cover a broader range of MDA concentration permitting evaluating also extremely higher saliva MDA value. Therefore 2 calibration points, “0.3 nmol/well” and “0.45 nmol/well” were added.

- 1. Sample pretreatment and optimization

The overall aim of the project was to find an assay method that would use a minimum of sample amount, be easy to implement and not time-consuming.

Parts 2.10.1 to 2.10.4 were carried out using a saliva pool consisting of a mixture of saliva samples collected from 10 individuals. Each condition was carried out over five replicates, due to the limited amount of saliva available. Parts 2.10.5 to 2.10.7 used samples from 3 other individuals to estimate the inter-individual effect of pre-treatments. In these sections, to monitor the fate of MDA during the preparative stage and evaluate a recovery yield, a sample corresponding to high concentration of MDA (1 µM) was mimicked.

Each salivary sample was collected from an in-house panel of healthy subjects in accordance with biosafety regulations for studies involving human subjects. Saliva was collected at rest in a collection jar placed on ice to preserve sample from inherent degradation. All samples were mixed, clarified or deproteinized, aliquoted in 2 mL cryotubes and frozen at -80°C for less than 1 month. The required volume was thawed just before each assay.

- - 1. *Study of the effect of the amount of sample used for the assay and the degree of sample concentration*

From the literature review, results obtained on the amount of native salivary MDA from non-pathological patients are very heterogeneous. Thus, the issue regarding the amount of saliva that has to be introduced into the wells for MDA detection was addressed by testing several volumes of saliva, i.e. 150 μL / 40 μL / 20 μL / 10 μL / 4 μL.

Moreover, saliva was concentrated by 2 and by 4 using a centrifugal evaporator. A volume of 40 μL of these concentrated saliva samples was introduced into the wells.

- - 1. *Study of the effect of sample clarification*

Clarification is a gravimetric centrifugation method in which large particles are removed. The supernatant, homogeneous and free of debris and large salivary proteins such as mucins, is analyzed.

We assessed the effect of clarification versus no treatment on the determination of native MDA in saliva. Saliva was centrifuged for 15 minutes at 15,000 g at 4°C. 40 μL of centrifuged or uncentrifuged saliva were deposited in each well, 135 μL of reagent and water completed until 300 μL. The rest of the protocol was the same as described in § 2.1.

- - 1. *Study of the effect of a detergent*

Sodium dodecyl sulfate (SDS) is an anionic surfactant. It can form micelles due to its amphiphilic properties. It can act thus as a detergent, breaking the covalent bonds of macro-molecules (proteins, lipids) and making the analyte (MDA) available for the assay reaction.

400 μL of saliva was added to 400 µL of 10% SDS solution. The mixture was vortexed for 5 minutes. The comparison was carried out on a non-centrifuged saliva sample. 80 µL of the saliva/SDS mixture were deposed per well. The rest of the protocol was the same as in § 2.1.

- - 1. *Sample deproteinization study*

Proteins are the main constituents of saliva (1 mg/mL), and can cause interference, notably due to the presence of bound sialic acid/N-acetyl-neuraminic acid (34, 53). Indeed, if present in the reaction medium, these compounds may fluoresce, hindering assay interpretation. TCA is often used to precipitate proteins from biological fluids. In the chemical method used in our protocol proteins are removed from the sample using a 10% TCA solution. The mixture was vortexed for 5 minutes, frozen for 12 hours and centrifuged the following day for 1 hour at 15,000 g at 4°C, with only the supernatant retained. 150 µL of the extract was inserted into the wells. The rest of the protocol was the same as in § 2.1.

- - 1. *Comparison of clarification versus sample deproteinization*

MDA (+/-) BHT until 280 µM was introduced into the sample and it was compared with the previous preparative methods. BHT addition was tested after preparation and before freezing if it had not been added with MDA, in order to evaluate the effect of its protective antioxidant role towards MDA in each preparative method.

- - 1. *Study of the antioxidant effect of BHT*

BHT was added until 280 µM to samples before or after clarification (§2.10.2). A control in the absence of BHT was carried out.

- - 1. *Study of the effect of sample freeze/thaw cycles*

Once the samples had been prepared, they were assayed before freezing and after a freeze/thaw cycle. To ensure that the preparation conditions tested corresponded to those used in future studies, the MDA concentration in samples that had undergone 2 freeze/thaw cycles was determined.

- 1. Verification of the specificity of the dosing method using the dose-response approach

To ensure that the method doses MDA and no other interacting product, it was necessary to implement a dose-response approach during development. This quantitative analysis technique was used to control and minimize matrix effects that interfere with analyte measurement signals. It involved adding known quantities of a standard solution of MDA to identical samples. The delta of fluorescence between the unspiked saliva sample and the blank corresponds to the equivalent MDA concentration of the sample if the linear calibration by least squares was achieved.

This method was very time-consuming for salivary samples, but was essential when developing methods for biological samples where matrix effects are present.

To implement this method, 150 µL of deproteinized saliva, known quantities of standard MDA solution, 135 µL of reagent solution and completed until 300 µL of ultrapure water were added to each well. Due to the small amount of saliva available, a 6-point dosing range was established with the following quantities added per well: 0; 0.006; 0.018; 0.03; 0.06; 0.09 nmol of MDA, with 2 replicates per quantity added.

This method was used throughout the development of the salivary sample pre-treatment method.

- 1. Choice of data processing method

Throughout the salivary sample tests, calculations were carried out using 3 different methods:

- Using the equation of the regression line of the calibration range (pure MDA) from the raw data

- Using the equation of the regression line of the calibration range (pure MDA) from data corrected for the blank value (solution without MDA)

- Using the equation of the regression line of the range of MDA additions into a saliva sample (pool of saliva from different individuals) used for the dose-response approach compared to a direct method.

1. Characterization and validation using the accuracy profile
   1. Validation data collection

The aim of the validation experimental design was to estimate the method's performance under conditions similar to those of its routine application. This technique enables us to determine how the method will perform when used on a regular basis. The tests had to be carried out using the method as it will be used routinely.

To achieve the plan, were carried out:

- 5 series of measurements (1 ≤ i ≤ 5) (i.e. 5 different assays) meaning reproducibility conditions which are the most stringent condition compare to repeatability conditions.

- For each series, there are 2 repetitions (1 ≤ j ≤ 2) (due to the low volume of saliva available) meaning repeatability conditions

- 7 concentration levels (1 ≤ k ≤ 7) for the calibration range and 6 concentration levels for the saliva samples (1 ≤ k ≤ 6) covering the range of application of the method.

- 1. Accuracy profile

The accuracy profile has been implemented in accordance with AFNOR NF-V03-110 and Feinberg et al. (55). The interval that contains on average a defined proportion (in %) of future measurements, obtained according to a given procedure and for a given concentration has a so-called tolerance limit, β. The so-called acceptability interval specifies the required performance for the method, expressed as an acceptable relative deviation (in %), called λ, around the reference value. Preliminary tests have established: β = 80% and λ = 30%. The range of validity of the method is determined by the zone of the accuracy profile in which the method provides a proportion of acceptable results at least equal to β. It is limited by a lower bound which is equivalent to the lower quantification threshold, determined by the intersection between the acceptability limit́ and the tolerance limit. The upper bound will be chosen as the highest concentration in the range. As soon as the tolerance interval falls outside the acceptability interval, it can be concluded that the method is no longer capable of providing sufficient so-called acceptable results with the choices made for λ and β. This graph also provides other indications: the variation in accuracy (with recovery) as a function of concentration. In addition, this approach makes it possible to model accuracy and uncertainty over a concentration interval.

The accuracy profile was implemented:

On one hand, on a pure MDA concentration range

On the other hand, on a concentration range of MDA added to 4 salivary samples corresponding to 4 different individuals for whom at least 3 mL of saliva had been collected.

1. Protocol validation


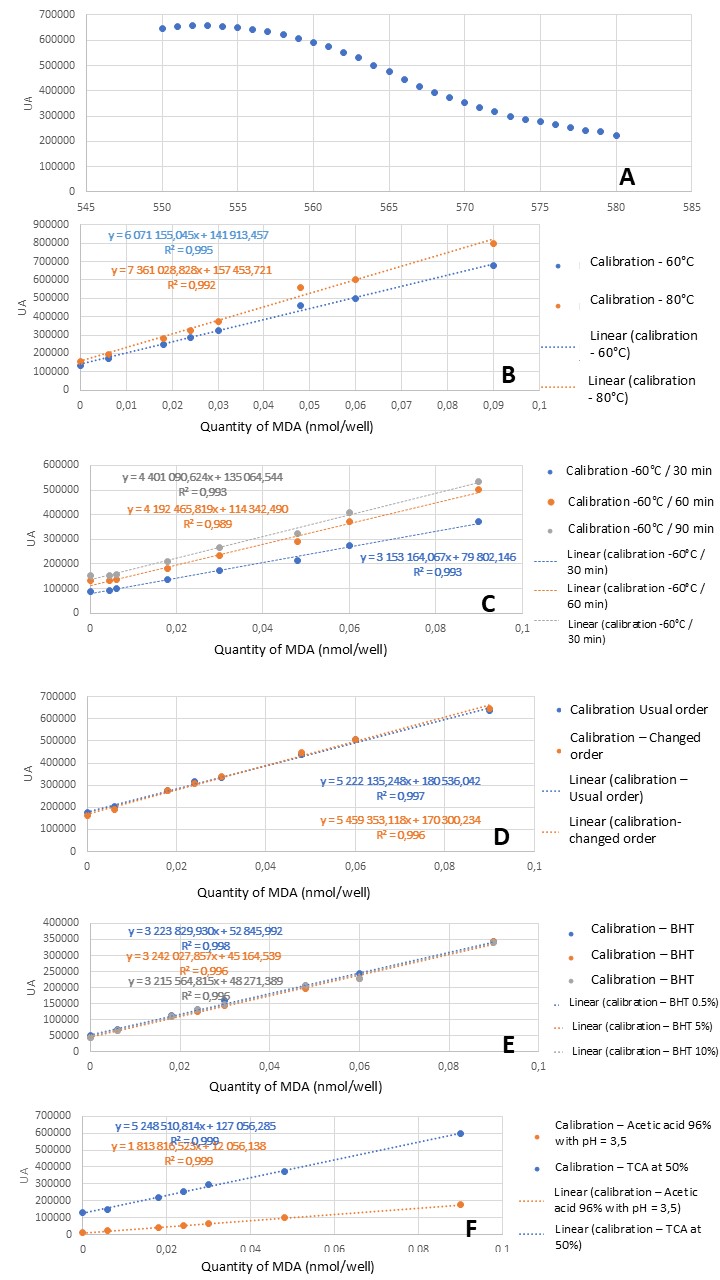


Figure 1: in all graph the measured values are represented by the dots and the regression line by the dotted line.

A: spectrum of the TBA:MDA adduct with λexc = 532 nm

B: calibration lines UA=f(QtyMDA) with comparison of incubation temperature

C: calibration lines UA=f(QtyMDA) with comparison of incubation time

D: calibration lines UA=f(QtyMDA) with comparison of order of reagent introduction: incubate BHT + TCA + TBA all together (blue) or premix BHT + TCA + sample incubated 15 minutes and then add TBA and incubate (orange)

E: calibration lines UA=f(QtyMDA) with comparison of BHT concentration

F: calibration lines UA=f(QtyMDA) with comparison of acid used in reagent

- 1. Procedure for developing the fluorometric method

Figure 1A shows the evolution of the emission spectrum of the TBA:MDA adduct with λexc = 532 nm.

The maximum fluorescence intensity is at a wavelength λem = 553 nm.

For the rest of the protocol development, we therefore chose λem = 553 nm in line with the literature (35).

The reaction in the absence of TBA adduction shows a negligible signal (Table 1) representing 1.6% of the fluorescence intensity value of the signal in the presence of TBA in the reaction medium.

|  | Fluorescence intensity (AU) if [MDA] = 0µM | |
| --- | --- | --- |
| replicates | without TBA | With TBA |
| 1 | 721 | 51429 |
| 2 | 856 | 50100 |
| 3 | 764 | 47859 |
| Mean | 780 | 49796 |
| ratio | 1,6% |  |

Table 1: Fluorescence values for reaction mixtures with and without TBA.

- 1. Study of the effect of incubation time and temperature

Figure 1B shows the calibration lines UA=f(QtyMDA) as a function of incubation temperature.

The linear regression modeling the reaction at 80°C has a directrix 1.2 times greater than that of the reaction at 60°C. This proportion was not very high, which validates the use of the 60°C assay. Indeed, the aim is to work with biological samples and over-oxidation of salivary compounds must be avoided. Thus the lowest possible temperatures allowing the TBA:MDA adduction reaction to take place must be used.

Figure 1C shows the calibration lines UA=f(MDAQty) as a function of incubation time.

It has been noted that the directing coefficient of each linear regression increased when incubation time increase. It means that higher the incubation temperature is, higher is the sensitivity of the assay for pure MDA. An incubation time of 30 minutes versus 60 minutes reduced reactivity by 25%. This reduction was judged as acceptable for further experimentation, the main aim of the protocol being to minimize oxidation of salivary components during the measurement. An incubation time of 30 minutes was therefore chosen for further experimentation. However, the y-intercept (corresponding to fluorescence intensity of sample-free wells) also increase when incubation time increase and is due to natural evolution of the reactional mixture.

- 1. Study of the effect of reagent introduction order

Figure 1D shows the calibration lines UA=f(QtyMDA) with comparison of the order of reagent introduction. The order of introduction of the reagents has no effect on the directing coefficient, nor on the y-intercept of the calibration line, since the equations of the calculated lines are identical.

In the following development, the reagent solution is carried out as in the initial protocol (§2.1), i.e. TBA, TCA and BHT added at the same time, followed by the incubation step.

- 1. Study of the effect of BHT concentration

Figure 1E shows the calibration lines UA=f(QtyMDA) comparing the use of BHT solutions at 3 different concentrations. There was no effect of BHT concentration on the directrix or y-intercept of the calibration line on standard solution range.

The effect has been verified with a salivary pool from healthy individual and the amount of equivalent MDA into saliva tend to be higher with 5% BHT than without BHT but the results were not significant in the saliva from healthy individual. Saliva of pathological individual known to have a higher MDA concentration could be more sensitive to parameter evolution.

Moreover, in accordance with literature, BHT avoid overoxidation during the heating step of the reaction with TBA in the biological sample measurement. For further development, we decided to keep the BHT concentration used in the initial protocol, i.e. 5% in the reagent.

- 1. Test of the acid to be used in the reagent solution

Figure 1F shows the calibration lines UA=f(QtyMDA) as a function of the acid used in the reagent.

The directrix of the calibration line with acetic acid reduced to pH 3.5 was 3 times lower, but also with a 10-fold lower intercept. The reaction mixture with TCA showed much better reactivity, and it was decided to retain it for further development.

- 1. Study of the effect of TCA concentration

The background noise (Figure 1F) was lower with acetic acid, indicating that the presence of TCA at a high final concentration (around 800 mM) induces a background noise that should be reduced by lowering the TCA concentration. The TCA solution was thus reduced to 9.8% instead of 50% in the preparation protocol, which corresponded to 150 mM in the final reaction medium, 5 times less than initially. By decreasing this concentration, the pH increased by nearly 2.5 pH units both before and after the reaction (Table 2). The drop in pH after incubation, in both situations, proved that a reaction indeed took place in each well.

|  | pH before incubation | pH after incubation | Blank value (UA) |
| --- | --- | --- | --- |
| TCA at 50% | 0,35 | -0,12 | 153 621 |
| TCA at 9,8% | 2,8 | 2,5 | 46 248 |

Table 2: Presentation of blank values and pH before and after incubation


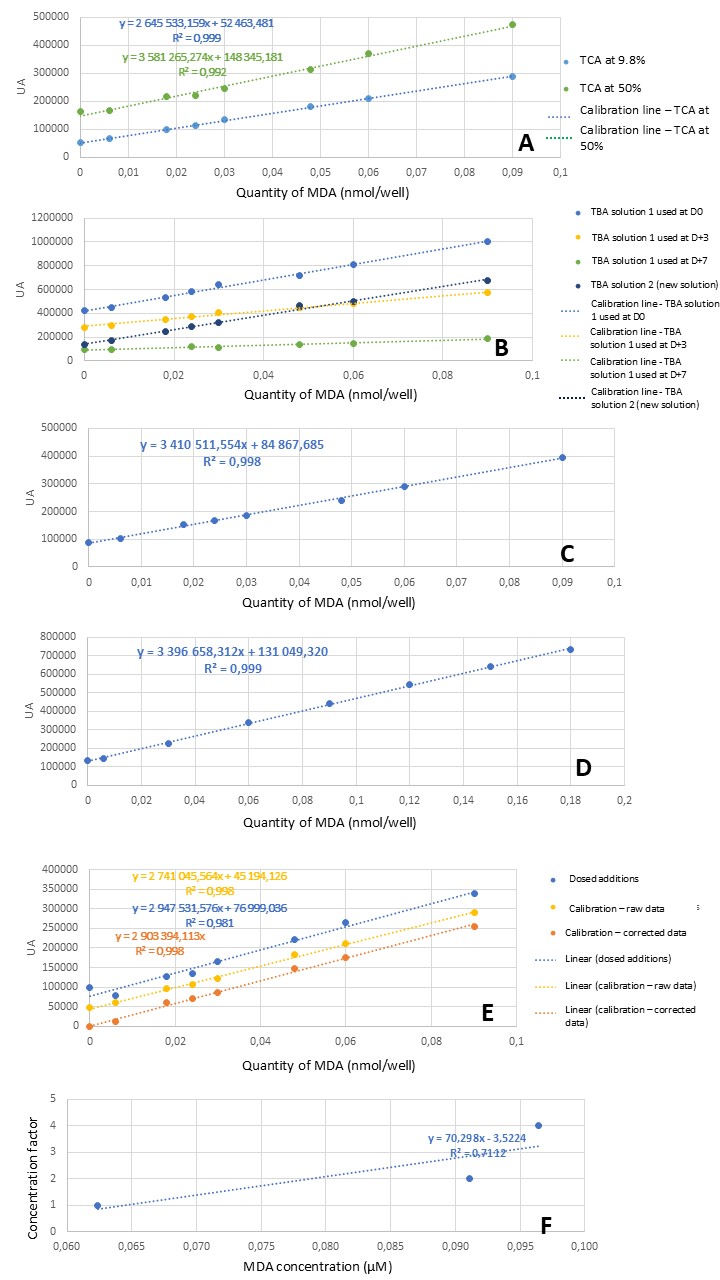

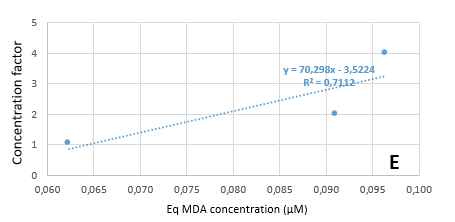


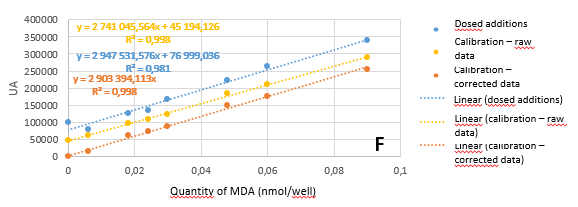


Figure 2: in all graph the measured values are represented by the dots and the regression line by the dotted line.

A: calibration lines UA=f(QtyMDA) with comparison of the TCA concentration used in the reagent

B: calibration lines UA=f(QtyMDA) with comparison of the TBA solutions used in the reagent at different time of storage (blue, yellow and green) and between 2 solutions freshly prepared (blue and dark blue)

C : calibration line UA=f(QtyMDA) ranging from 0 to 0.09 nmol MDA/well

D : calibration line UA=f(QtyMDA) ranging from 0 to 0.18 nmol MDA/well.

E: calibration line Concentration factor=f(eq MDA concentration) ranging from 1 to 4.

F: calibration lines (yellow and orange) UA=f(QtyMDA) and dosed addition line (blue) UA=f(QtyMDAdded) for assay n°1

Figure 2A presents the calibration curves of UA=f(MDA amount) in each reaction medium with the two TCA concentrations used to prepare the reagent. Additionally, by using a lower concentration of TCA, it was possible to reduce the y-intercept value by nearly 30%, thus decreasing the background noise. Since the obtained curves were parallel, no variation in reactivity with the change in TCA concentration was observed. Only the blank value decreased with the concentration reduction, proving that TCA induced fluorescence in the reaction medium.

For further development, we therefore decided to use a 9.8% TCA solution in the reagent.

- 1. Stability of reagents

Figure 2B presents the calibration curves of UA=f(MDA amount) based on TBA solutions used at different time of storage. The TBA solution degraded over time as a decrease of the directing coefficient of each linear regression over the days (D+O, D+3, D+7) was observed with TBA solution 2 was freshly prepared, the directing coefficient of linear regression is the same than solution 1 at D+0 ;

It was decided that the TBA solution should be stored for a maximum of 48 hours in the refrigerator.

The figure 3 summarizes the step-by-step approach.


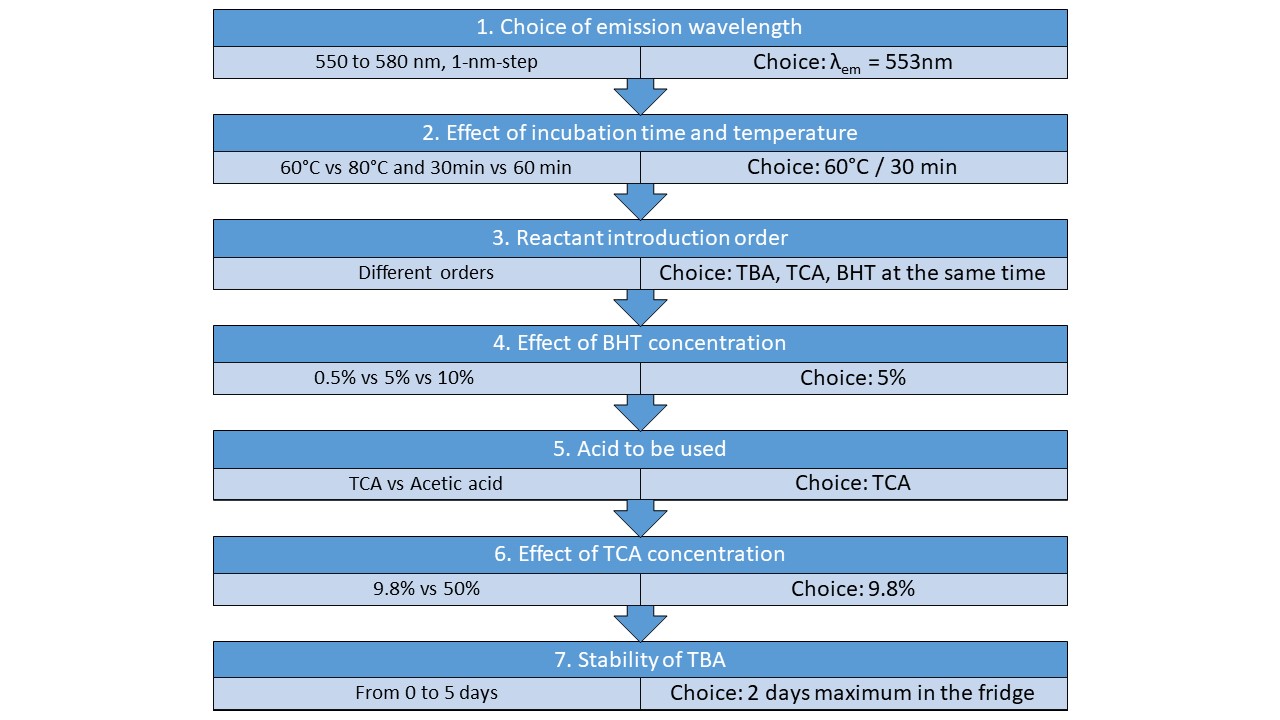


Figure 3: step-by-step approach of the analytical method.

- 1. Verification of the measurement range
     1. Range from 0 to 0.09 nmol/well

Figure 2C presents the calibration curve of UA=f(MDA amount) ranging from 0 to 0.09 nmol of MDA/well.

The linear correlation coefficient R² was greater than 0.99. Amounts of MDA and corresponding fluorometric responses are highly correlated.

- - 1. Range from 0 to 0.18 nmol/well

Figure 2D presents the calibration curve of UA=f(MDA amount) ranging from 0 to 0.18 nmol of MDA/well.

The linear correlation coefficient R² is greater than 0.98. Amounts of MDA and corresponding fluorometric responses are highly correlated.

The method can therefore be used for the assay of saliva samples collected from pathological subjects.

- 1. Optimization of Sample Pretreatment

When the expression 'unusable results' is used, it means that the fluorescence intensity was equal to that of the blank for the saliva samples. This indicates that MDA was not detected in the sample.

- - 1. Study of the Effect of Dilution and Concentration of Saliva (without Deproteinization)

Figure 2E presents the results obtained with the modified protocol with the following parameters: reagent prepared with 9.8% TCA and deproteinization of the saliva sample (section M&M 2.9.4). There was no proportionality between the MDA concentration of the saliva sample; however, there was only a trend towards linearity (R²=0.71). Indeed, concentration by evaporation changed the physical properties of the saliva. The fluid thickened, and the concentration increased the turbidity, which is incompatible with the chosen detection mode during the assay. Similarly, a diluted sample did not have the same refractive index as an undiluted sample, making it impossible to compare the measurements. Thus, with a set of samples having a wide range of MDA content, highly concentrated saliva samples could not be diluted to be included in the calibration range. The calibration range will need to be extended with higher concentrations.

- - 1. Study of the Effect of Clarification

This study is not presented because the results could not be exploited.

- - 1. Study of the Effect of a Detergent: SDS (without Deproteinization)

This study is not presented because the results could not be exploited.

- - 1. Study of Specificity by the dose-response approach

Figure 2F presents the calibration curves (yellow and orange for raw and corrected data respectively) UA=f(MDA amount) and the spiked addition curve (blue) UA=f(added MDA amount). The spiked addition method allows checking that the linear regression curve after spiked additions of MDA in a saliva sample had a slope equivalent to the regression curve of a pure MDA solution calibration range. The smaller the difference between these two slopes, the better the assay accuracy and analyte extraction during the sample pretreatment. The results meet this condition since 1% variation was observed between the slope of the calibration range and the slope of the spiked saliva sample range. Moreover, the slope of the spiked addition curve was 1.07 times greater than that of the calibration curve, considering the raw data, and 1.01 times greater when considering the corrected data. The deproteinization of saliva proved to be effective and was retained for the preparation of saliva samples.

- - 1. Comparison of Clarification versus Deproteinization of the Sample

The four experiments showed that the introduced MDA concentration was recovered after clarification only. After protein depletion with TCA, only 4 to 25% of the initial MDA concentration was recovered (3 intermediate preparative experiment conducted on 3 different days, 2 replicates, n= 3 subjects). One possible interpretation is that MDA binds to and/or is trapped by the sample proteins to buffer this oxidative excess. Although the results of these two methods were usable, we chose to proceed with clarification to implement the analytical method, considering the practical constraints of the studies while being aware of the potential interactants, which are the salivary proteins.

- - 1. Study of the Antioxidant BHT

If BHT was not added, the amount of MDA recovered in the sample after clarification varied from 1 to 100% of the introduced amount (one experiment day, 2 replicates, n = 3 subjects) meaning that the recovery percentage depended on the sample and was not reliable. If BHT was introduced after clarification, the amount of MDA presented in the sample was 50 to 75% of the initially introduced amount (one experiment day, 2 replicates, n = 3 subjects) even though the recovery was not total it is similar and repeatable among the samples. BHT seemed to play an antioxidant role, especially when introduced quickly into the samples. Therefore, BHT had to be introduced before clarification to maximize the amount of MDA recovered in the sample.

- - 1. Study of the Effect of Freeze-Thaw Cycles on Samples

72 to 80% of the added amount is recovered in samples that underwent a freeze-thaw cycle compared to those that did not (one experiment day, 2 replicates, n = 3 subjects). If the preparation and assay were done on samples that had already undergone a freeze-thaw cycle (2 experiment days, 2 replicates, n = 3 subjects), the initially introduced MDA amount was also recovered.

- 1. Conclusions about preparation

The practice of saliva collection during clinical and sensory studies does not allow for immediate preparation and assay. Therefore, BHT must be added, and the samples must be clarified after undergoing a freeze-thaw cycle, then refrozen in microplates, and thawed again to perform all analyses simultaneously.

Analysis is designed to be performed in 96 wells microplates. Two Internal Quality Control in replicate were added into each one. It consisted of a saliva sample that had undergone the same preparatory steps as the study samples and is placed in each microplate to control a bias between plates.

The sample preparation protocol that optimized MDA yield considering the study conditions and context is presented in Figure 4.


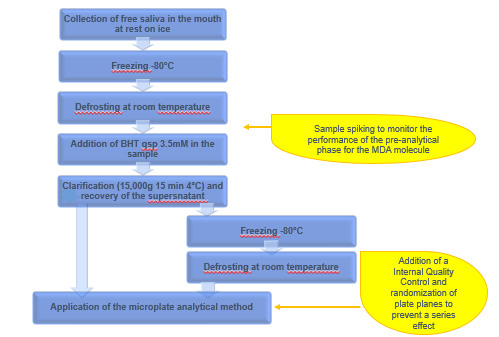


Figure 4: optimized sample MDA yield considering the study conditions and context

1. Verification of the Concentration of an Average Saliva Sample by the Spiked Addition Method


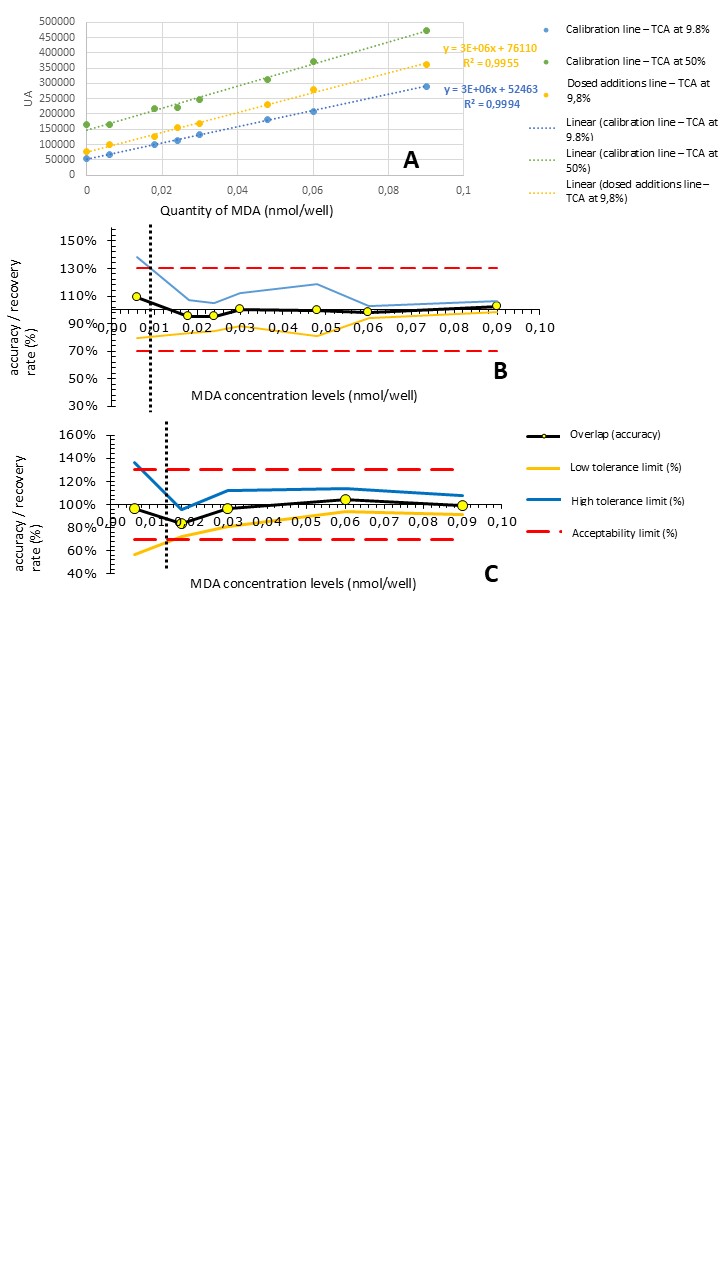

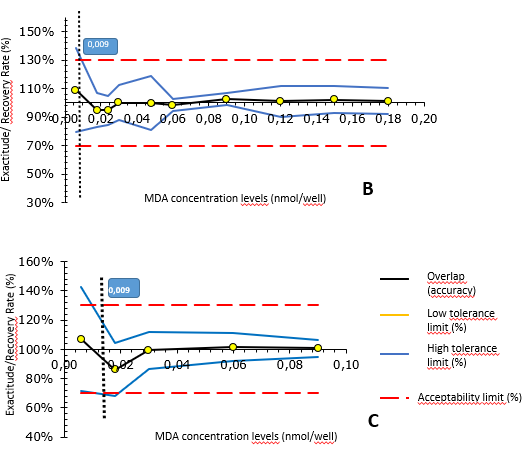


Figure 5:

A: UA=f(QtyMDA) calibration lines as a function of different TCA solutions. The measured values are represented by the dots and the regression line by the dotted line.

B: Accuracy profile obtained for standard solutions range

C: Accuracy profile obtained for a saliva sample from an individual spiked with MDA at 5 concentration levels.

Figure 5A presents the calibration curves (blue and green) UA=f(MDA amount) and the spiked addition curve (yellow) UA=f(added MDA amount).

As previously noted, it was necessary for the spiked addition curve to be above the calibration curve to obtain usable results. Using a solution highly concentrated in TCA in the reagent caused too high background noise (sections 6 and 9 of the Results) and made it impossible to use the spiked addition method, as it was then below the calibration curve on the graph. By reducing the TCA concentration from 50% to 9.8%, the blank value decreased, and graphically, the spiked addition curve passed above the calibration curve, making the results exploitable.

Plotting the spiked addition curve ensured that it had a slope very close to that of the pure analyte calibration curve (3.106 UA/nmol). The difference in the y-intercept was explained by the presence of MDA in the saliva sample.

The regression coefficient R² was > 0.99, indicating excellent regression quality, matching the quality level of a pure analyte calibration curve.

1. Determination of the Quantification Limit and Characterization by Accuracy Profile

Figure 5B shows the accuracy profile obtained at different levels of the pure analyte concentration in solution using the direct method with raw data (see section 2.12.). Thanks to the accuracy profile representation, the quantification limit was determined as the intersection of the vertical dashed line with the x-axis. It was equal to 0.0092 nmol/well, corresponding to an MDA concentration of 0.031 µM.

The direct method using corrected data (see section 2.12.) presented a higher quantification limit (0.016 nmol/well, or 0.053 µM) than the one using raw data. It was decided to proceed with direct calibration using raw data because this method provided a lower quantification limit compared to the method using data corrected for the blank value

Figure 5C shows the accuracy profile obtained for a saliva sample from an individual that had undergone preparative steps as described (see sections *2.10.6* and 2.11) spiked with MDA at 5 concentration levels.

The determination of the quantification limit with spiked Addition Method was carried out as previously described.

It was 0.009 nmol/well, corresponding to an MDA concentration of 0.031 µM. Thanks to the preparative process set up, the accuracy of the method was at the same level than the standard.

1. Application of the dose-response approach compared to a direct method

The dose-response approach is not suitable for measuring saliva samples in microvolumes for a research project. Indeed, the method as implemented during development (6 concentrations and 2 replicates) required a volume of saliva of 1.8 mL per sample. This volume may be difficult or impossible to obtain in the case of pathological subjects for example. In case of the direct method only 150 µL of prepared supernatant is required by replicate and is convenient for low volume saliva samples.

1. Application of the developed Protocol in individuals in experimental research conditions


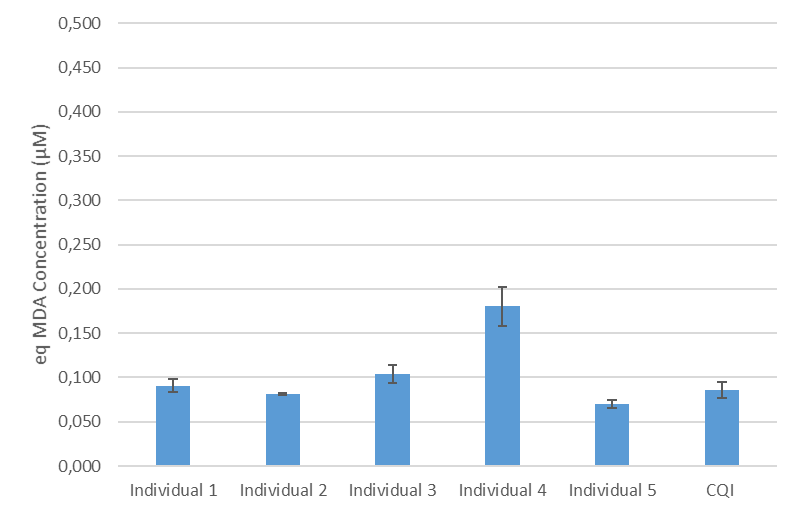


Figure 6. Salivary MDA concentration in 5 individuals (in µM eq MDA) from 2 repeated measurements.

|  | Individual 1 | Individual 2 | Individual 3 | Individual 4 | Individual 5 | CQI |
| --- | --- | --- | --- | --- | --- | --- |
| Assay 1 (operator 1, day 1, plate 1) = repetition 1 | 0.098 | 0.082 | 0.114 | 0.202 | 0.074 | 0.095 |
| Assay 1 (operator 1, day 1, plate 2) = repetition 2 | 0.084 | 0.080 | 0.093 | 0.158 | 0.066 | 0.077 |
| Mean | 0.091 | 0.081 | 0.104 | 0.180 | 0.070 | 0.086 |
| Standard deviation | 0.007 | 0.001 | 0.010 | 0.022 | 0.004 | 0.009 |
| Coefficient of variation | 8% | 1% | 10% | 12% | 6% | 10% |

Table 3. Data of MDA concentration of 5 saliva samples from 2 repeated measurements (in µM eq MDA).

Saliva sample from 5 individual were collected following experimental conditions adapted for studies involving the human person. It corresponds to repeatability conditions consisting in carrying out a single assay to compare all the samples from the same project

Samples were immediately frozen and stored at -80°C during one year. Then, samples were defrozen at 20°C, BHT was introduced until 3.5mM into each sample. They were clarified by centrifugation 15 minutes at 15 000g at 4°C, supernatant was recovered and frozen at -80°C until analysis of all was perform in the same time.

The experimental plan of the analysis is detailed in Table 1.

Analysis were carried out and calculation was done using direct method and the calibration directrix from the raw data (as detailed in Protocol).

The results are presented in Figure 6. The MDA concentration are from 0.07 µM to 0.18 µM eq MDA with a range of coefficient of variation from 1 to 12% between the 2 replicates in 5 individuals. In this population sample, the mean of concentration is 0.11µM± 0.05 eq MDA.

References:

17: D Del Rio, AJ Stewart, N. Pellegrini

A review of recent studies on malondialdehyde as toxic molecule and biological marker of oxidative stress Nutr Metab Cardiovasc Dis, 15 (4) (2005), pp. 316-328

18: Chellouai Z, Moussaoui R, Benaissa S, Nachi M, editors. Le dosage du malondialdéhyde (MDA) par chromatographie liquide: Aspects pré- analytiques et analytiques. Journées de l'Innovation en Biologie; 2019.

19: HS Shin, DG. Jung

Sensitive Analysis of Malondialdehyde in Human Urine by Derivatization with Pentafluorophenylhydrazine then Headspace GC–MS

Chroma, 70 (2009), pp. 899-903

20: D Tsikas, S Rothmann, JY Schneider, M-T Suchy, A. Tretin

Development, validation and biomedical applications of stable-isotope dilution GC-MS and GC-MS/MS techniques for circulating malondialdehyde (MDA) after pentafluorobenzyl bromide derivatization: MDA as a biomarker of oxidative stress and its relation to 15(S)-8-iso-prostaglandin F2α and nitric oxide (NO)

J Chromatogr B Analyt Technol Biomed Life Sci, 1019 (2016), pp. 95-111

21: R Malaei, AM Ramezani, G. Absalan

Analysis of malondialdehyde in human plasma samples through derivatization with 2,4-dinitrophenylhydrazine by ultrasound-assisted dispersive liquid-liquid microextraction-GC-FID approach

J Chromatogr B Analyt Technol Biomed Life Sci, 1089 (2018), pp. 60-69

22: C Syslovà, P Kacer, M Kuzma, V Najmanová, Z. Fenclová

Rapid and easy method for monitoring oxidative stress markers in body fluids of patients with asbestos or silica-induced lung diseases

J Chromatogr B Analyt Technol Biomed Life Sci, 877 (2009), pp. 2477-2486

23: R Mendonca, O Gning, C Di Cesare, L Lachat, NC Bennett, F Helfenstein, *et al.*

Sensitive and selective quantification of free and total malondialdehyde in plasma using UHPLC-HRMS

J Lipid Res, 58 (9) (2017), pp. 1924-1931

24: P Hannan, J Khan, Z Iqbal, I. Ullah

Simultaneous Determination of Endogenous Antioxidants and MDA by RPHPLC Coupled with Electrochemical Detector in Serum Samples

J Liq Chromatogr Relat Technol, 38 (2015), pp. 1052-1060

25: Z Rezaei, A Jamshidzadeh, E Sanati

A rapid and sensitive method for the determination of malondialdehyde as its hydralazine derivative in human plasma using high performance liquid chromatography

Anal Methods, 5 (2013), pp. 2995-2999

26: B Kim, W Jung, Y. Kho

Quantification of Malondialdehyde in Human Urine by HPLC-DAD and Derivatization with 2,4 Dinitrophenylhydrazine, 38, Kor Chem Soc (2017), pp. 642-645

27: D Hoyland, A. Taylor

A review of the methodology of the 2-thiobarbituric acid test

Food and chemical toxicology: an international journal published for the British Industrial Biological Research Association, 40 (1991)

271-*91

28: M Richard, P Guiraud, J Meo, A. Favier

High-performance liquid chromatographic separation of malondialdehyde—thiobarbituric acid adduct in biological materials (plasma and human cells) using a commercially available reagent

J Chromatogr, 577 (1992), pp. 9-18

31: R Kanďár, V Mužáková, Čegan A.Highly Specific

Simple and Rapid Method for the Determination of Malondialdehyde in Blood Using High-Performance Liquid Chromatography

Clinical chemistry and laboratory medicine, 40 (2002), pp. 1032-1035

32: I Young, E Trimble

Measurement of malondialdehyde in plasma by high performance liquid chromatography with fluorimetric detection

Annals of Clinical Biochemistry, 28 (1991), pp. 504-508

33: MA Carbonneau, E Peuchant, D Sess, P Canioni, M. Clerc

Free and bound malondialdehyde measured as thiobarbituric acid adduct by HPLC in serum and plasma

Clinical chemistry, 37 (8) (1991), pp. 1423-1429

34: SH Wong, JA Knight, SM Hopfer, O Zaharia, CN Leach Jr., FW Sunderman Jr.

Lipoperoxides in plasma as measured by liquid-chromatographic separation of malondialdehyde-thiobarbituric acid adduct

Clinical chemistry, 33 (2 Pt 1) (1987), pp. 214-220

35: H Ohkawa, N Ohishi, K. Yagi

Assay for lipid peroxides in animal tissues by thiobarbituric acid reaction

Anal Biochem, 95 (2) (1979), pp. 351-358

36: MJ Richard, B Portal, J Meo, C Coudray, A Hadjian, A. Favier

Malondialdehyde kit evaluated for determining plasma and lipoprotein fractions that react with thiobarbituric acid

Clinical chemistry, 38 (5) (1992), pp. 704-709

37: C Jo, DU. Ahn

Fluorometric analysis of 2-thiobarbituric acid reactive substances in turkey

Poult Sci, 77 (3) (1998), pp. 475-480

38: D. Armstrong

Free radical and antioxidant protocols. 108

Humana Press, Totowa, NJ, USA (1998), pp. 170-171

39: G. Carre

Compréhension des mécanismes lors de la photocatalyse appliquée à la dégradation des microorganismes: application au traitement de l'air et aux textiles auto-décontaminants

Université de Strasbourg (2013)

42: K. Yagi

Simple Assay for the Level of Total Lipid Peroxides in Serum or Plasma. Free Radical and Antioxidant Protocols Methods in Molecular Biology. 108

Humana Press, Totawa, NJ, USA (1998), pp. 101-106

43: S Hedrei Helmer, A Kerbaol, P Aras, C Jumarie, M Boily

Effects of realistic doses of atrazine, metolachlor, and glyphosate on lipid peroxidation and diet-derived antioxidants in caged honey bees (Apis mellifera)

44:
C. Landry

Évaluation de l’état du système redox et des rétinoïdes comme biomarqueurs chez la perchaude en lien avec le déclin de la population du lac Saint-Pierre

Université du Québec, Montreal (2017)

45: JA Buege, SD. Aust

Microsomal lipid peroxidation

Methods Enzymol, 52 (1978), pp. 302-310

46: D Armstrong, R. Browne

The analysis of free radicals, lipid peroxides, antioxidant enzymes and compounds related to oxidative stress as applied to the clinical chemistry laboratory

Adv Exp Med Biol, 366 (1994), pp. 43-58

47:
P Maboudou, D Mathieu, H Bachelet, JF Wiart, M. Lhermitte

Detection of oxidative stress. Interest of GC-MS for malondialdehyde and formaldehyde monitoring

Biomed Chromatogr, 16 (3) (2002), pp. 199-202

48: Z Singh, P Karthigesu, P Singh, R. Kaur

Use of Malondialdehyde as a Biomarker for Assessing Oxidative Stress in Different Disease Pathologies: a Review

Iran J Public Health, 46 (supple 3) (2015), pp. 7-16

49: M Khoubnasabjafari, K Ansarin, A. Jouyban

Salivary malondialdehyde as an oxidative stress biomarker in oral and systemic diseases

J Dent Res Dent Clin Dent Prospects, 10 (2) (2016), pp. 71-74

50: L Tothova, N Kamodyova, T Cervenka, P. Celec

Salivary markers of oxidative stress in oral diseases

Front Cell Infect Microbiol, 5 (2015), p. 73

51: D. Armstrong

Free radical and antioxidant protocols. Introduction

Methods Mol Biol (1998), p. 108

p. v-viii

52: G Camejo, B Wallin, M. Enojärvi

Analysis of Oxidation and Antioxidants Using Microtiter Plates

D Armstrong (Ed.), Free Radical and Antioxidant Protocols Methods in Molecular Biology™, Humana Press (1998), p. 108

53: G Lepage, G Munoz, J Champagne, CC. Roy

Preparative steps necessary for the accurate measurement of malondialdehyde by high-performance liquid chromatography

Anal Biochem, 197 (2) (1991), pp. 277-283

54:
R Kaur, Z Singh, IP Karthigesu, P. Singh

Use of Malondialdehyde as a Biomarker for Assessing Oxidative Stress in Different Disease Pathologies: a Review

Iran J Publ Health, 43 (2015), pp. 7-16

55: Feinberg M. Interpretation of the Accuracy Profile. Le Cahier des techniques de l'INRA. 2010;Special Volume:45-60.
